# Andrographolide inhibits STAT1 expression and desensitizes interferon γ-induced priming in macrophage cells

**DOI:** 10.3389/fphar.2026.1855424

**Published:** 2026-09-03

**Authors:** Junjun Liu, Fangfang Liu, Xiaoyu Hu, Xiaopei Cui, Jie Wang, Qian Cai, Chun-Guang Li, Wen Cheng, Fan Jiang

**Affiliations:** 1 Gerontology and Anti-Aging Research Laboratory, Department of Geriatric Medicine, Qilu Hospital of Shandong University, Jinan, Shandong, China; 2 Department of Cardiology, Shandong Medical and Pharmaceutical University Hospital, Binzhou, Shandong, China; 3 Department of Cardiology, Qilu Hospital of Shandong University, Jinan, Shandong, China; 4 NICM Health Research Institute, Western Sydney University, Westmead, NSW, Australia; 5 Department of Traditional Chinese Medicine, Qilu Hospital of Shandong University, Jinan, Shandong, China

**Keywords:** andrographolide, anti-inflammatory effect, macrophage, priming, STAT1, type I interferon, type II interferon

## Abstract

**Background:**

The herbal medicine *Andrographis paniculata* has broad anti-inflammatory effects. In the present study, we aimed to clarify whether andrographolide (And), neoandrographolide (Neo), and deoxyandrographolide (Deo), three of the most abundant bioactive diterpene lactone compounds in *A. paniculata*, can modulate the pro-inflammatory priming effects of interferon (IFN)-γ in macrophage cells.

**Methods:**

The human monocytic cell line THP-1 was pre-activated with lipopolysaccharide (LPS) or phorbol 12-myristate 13-acetate (PMA). Murine and human primary macrophage cells were pre-activated with LPS.

**Results:**

In THP-1 cells, we showed that both LPS and PMA stimulated the expression of signal transducer and activator of transcription 1 (STAT1), the pivotal signaling module in type I and II IFNs; these responses were blunted by And at non-cytotoxic concentrations but not by Neo or Deo. In parallel, And significantly inhibited the priming effects of IFN-γ, including upregulation of the pro-inflammatory chemokine monocyte chemoattractant protein-1 and the NADPH oxidase subunit gp91phox. Mechanistically, we showed that the inhibitory effects of And on STAT1 expression in both LPS- and PMA-activated macrophages were, at least partly, attributable to the reduction in IFN-β expression, but not related to changes in IFN-β-induced activation of the transcription factor complex IFN-stimulated gene factor 3 or the stability of STAT1 mRNA. We confirmed the inhibitory effects of And on STAT1 expression and IFN-γ-induced priming in murine or human primary macrophages.

**Conclusion:**

Our results suggest that And may desensitize macrophage cells to IFN-γ-induced priming by inhibiting STAT1 expression. This effect of And may partly explain the multiple anti-inflammatory actions of this compound.

## 1 Introduction

Macrophage-mediated inflammatory reactions are implicated in a variety of acute and chronic inflammatory diseases of both infectious and non-infectious origin (McGonagle et al., 2021; Austermann et al., 2022; Hegarty et al., 2023; Chen et al., 2024; Wang et al., 2024). Maximum pro-inflammatory activation of the macrophage cells requires interferon (IFN)-γ-induced priming (also known as M1 polarization), which is characterized by increases in the expression of inducible nitric oxide synthase (iNOS/NOS2), the superoxide-generating NADPH oxidase, and various pro-inflammatory cytokines (Schroder et al., 2004; Schroder et al., 2006; Hu et al., 2008; Casbon et al., 2012; Ivashkiv, 2018; De Benedetti et al., 2021). Priming also enhances macrophages’ phagocytosis and antigen presentation functions. IFN-γ is mainly produced by natural killer cells, T helper 1 cells, and CD8^+^ cytotoxic T cells, following activation of pattern recognition receptors (PRRs) or stimulation by interleukin (IL)-12 (Ivashkiv, 2018). The signaling process downstream of the activated IFN-γ receptor (comprising the IFNγR1 and IFNγR2 subunits) is mediated by signal transducer and activator of transcription 1 (STAT1), which is phosphorylated by the receptor-associated protein tyrosine kinases Janus kinase 1 (JAK1) and JAK2. Phospho-STAT1 forms homodimers, which translocate to the nucleus and bind to the GAS (IFN-γ activation site) motif, promoting the transcription of interferon-stimulated genes (ISGs) (Ivashkiv, 2018). Accordingly, evidence supports that cellular responsiveness to INF-γ is partly determined by the expression level of STAT1 (Hu et al., 2002).

A key regulator of STAT1 transcription is type I IFN, principally IFN-α and IFN-β; low picomolar concentrations of IFN-β are sufficient to increase STAT1 expression (Ivashkiv and Donlin, 2014). Ligand binding of the IFN-α/β receptor (IFNAR, comprising the IFNAR1 and IFNAR2 subunits) recruits and activates JAK1 and tyrosine kinase 2, which, in turn, phosphorylate STAT1 and STAT2, leading to STAT1–STAT2 dimerization and nuclear translocation. In the nucleus, the STAT1/STAT2 dimer binds to IFN-regulatory factor 9 (IRF9), forming a transcriptional regulatory complex called IFN-stimulated gene factor 3 (ISGF3), which then binds to the IFN-stimulated response element (ISRE) present in the promoter regions of several hundred ISGs, including STAT1 (Ivashkiv and Donlin, 2014; McNab et al., 2015). Hence, STAT1 itself is a type I IFN target gene, the transcription of which is driven by several ISRE motifs in its promoter region (Amalraj et al., 2013; Yuasa and Hijikata, 2016). Consistent with this notion, it has been suggested that the pro-inflammatory priming effects of IFN-γ may be amplified by type I IFNs via the regulated expression of STAT1 (Gough et al., 2010). Type I IFNs are mainly produced by dendritic cells and monocytes/macrophages upon stimulation of PRRs (López De Padilla and Niewold, 2016). Therefore, in lipopolysaccharide (LPS)-stimulated macrophages, the upregulated STAT1 expression is attributable to IFN-β induction via the toll-like receptor 4 (TLR4) signaling, acting in an autocrine manner (Sheikh et al., 2014).

Several natural products have been shown to have inhibitory effects on the type I IFN system; the pharmacological mechanisms underlying these effects may be related to (1) inhibition of the production of IFN-α/β and (2) inhibition of IFNAR-triggered signal transduction (*e.g.*, phosphorylation of JAK1 or STAT1 (see Li et al. (2023)). In our pilot screening studies, we tested potential anti-inflammatory effects of a panel of medicinal herbs used in traditional Chinese medicine, using LPS-stimulated macrophages as a platform. We found that *Andrographis paniculata* (AP) exhibited a significant inhibitory effect on STAT1 expression. The dried aerial parts of AP are used ethnically in Chinese medicine for clearing “heat” and relieving “toxicity,” and are prescribed for treating flu- and common cold-associated symptoms such as fever, sore throat, and cough (Jiang et al., 2021). In other Asian countries, AP is also used to treat diabetes mellitus, malaria, and inflammatory diseases (Akbar, 2011; Kumar et al., 2021). The major bioactive constituents in AP are various diterpene lactones (Agrawal and Nair, 2022). In the present study, therefore, we aimed to clarify whether andrographolide (And), neoandrographolide (Neo), and deoxyandrographolide (Deo), three of the most abundant diterpene lactone compounds in AP, can modulate STAT1 expression and the pro-inflammatory priming effects of IFN-γ in macrophage cells.

## 2 Materials and methods

### 2.1 Reagents

LPS (#L2880) was from Merck KGaA (Darmstadt, Germany). Phorbol 12-myristate 13-acetate (PMA) (#HY-18739), hesperidin (#HY-15337), actinomycin D (#HY-17559), BAY 11–7,082 (#HY-13453), and baricitinib (#HY-15315) were from MedChemExpress (Monmouth Junction, New Jersey, United States). And (#ST01330120), Neo (#ST05030120), and Deo (#ST82560110) were from Nature Standard (Shanghai, China). IFN-β (#RP02774) and IFN-γ (#RP01038) were from ABclonal (Wuhan, Hubei Province, China). Tumor necrosis factor (TNF)-α (#DC008) was from Novoprotein (Suzhou, Jiangsu Province, China).

### 2.2 Cell culture

The human monocytic leukemia cell line THP-1 (#ZQ0086) and human embryonic kidney cell line HEK-293T (#ZQ0033) were purchased from Zhong Qiao Xin Zhou Biotechnology (Shanghai, China). THP-1 cells were cultured in RPMI-1640 medium (#C11875500BT, from Thermo Fisher Scientific, Waltham, MA, United States) supplemented with 10% fetal bovine serum (#BMC1020, from Abbkine, Wuhan, Hubei Province, China) and the antibiotic mixture of 100 U/mL penicillin and 100 μg/mL streptomycin (#15140122, Thermo Fisher). HEK-293T cells were grown in Dulbecco’s modified Eagle medium (DMEM) (#ZQ-100, from Zhongqiao Xinzhou Biotechnology) containing 10% fetal bovine serum (FBS) and antibiotics. All cells were maintained at 37 °C in a humidified atmosphere with 5% CO_2_.

Isolation and culture of human primary peripheral blood mononuclear cells were carried out as described in our previous publication (Dai et al., 2024). The protocol was approved by the Human Ethics Committee of Shandong University Cheeloo College of Medicine. Informed consent was obtained before commencement of the study. In brief, peripheral blood mononuclear cells from 20 mL of peripheral venous blood from healthy volunteers were purified using a Ficoll-based reagent kit from TBD Science (#LTS1077, Tianjin, China) and maintained in RPMI-1640 with 10% FBS and antibiotics. Macrophage differentiation was induced by incubation with 20 ng/mL human granulocyte–macrophage colony-stimulating factor (GM-CSF) (#RP00094, from ABclonal). Adherent macrophages were used for experimentation 7 days after induction.

Isolation and culture of murine primary bone marrow cells were performed according to our previous publication (Dai et al., 2018). The protocol was approved by the Institutional Animal Ethics Committee of Shandong University Cheeloo College of Medicine. Animals were handled in accordance with the National Institutes of Health Guide for the Care and Use of Laboratory Animals (NIH Publications No. 8023, revised 1978). Healthy male C57BL/6 mice were purchased from Vital River Laboratory (Beijing, China) and humanely killed by intraperitoneal injection of an overdose of pentobarbital sodium. Bone marrow cells were obtained by flushing the femur and tibia with sterile Hanks balanced salt solution. Red blood cells were lysed with 0.8% ammonium chloride. The remaining cells were maintained in RPMI-1640 with 10% FBS and antibiotics. Macrophage differentiation was induced by treating with macrophage colony-stimulating factor (M-CSF) (from BioLegend, San Diego, CA) at 20 ng/mL for 7 days. All primary cell cultures were maintained at 37 °C with 5% CO_2_.

### 2.3 Real-time PCR

Total RNA was extracted using TRIzol reagent (#15596018 from Thermo Fisher). RNA (2 μg for each sample) was reverse transcribed into cDNA using PrimeScript RT Reagent Kit (#RR037A from TaKaRa, Kyoto, Japan). Real-time PCR was performed using UltraSYBR Mixture (#CW0957H from CWBIO, Taizhou, Jiangsu Province, China) with a real-time PCR analyzer (Model C6) from TargetingOne (Beijing, China). The Ct (cycle threshold) values of target genes were normalized to those of the housekeeping gene GAPDH (glyceraldehyde-3-phosphate dehydrogenase). The relative gene expression levels were calculated using the 2^−ΔΔCT^ method. The primer sequences used were listed in Supplementary Table S1.

### 2.4 Western blot

Total protein was extracted in a lysis buffer (#P0013B from Beyotime Biotechnology, Shanghai, China) supplemented with 1 mM phenylmethylsulfonyl fluoride (#P0100 from Solarbio, Beijing, China) and a proteinase inhibitor cocktail (#CW2383S from CWBIO). Protein concentrations were determined using a protein assay kit (#KTD3001 from Abbkine). Equal amounts of protein (20 μg) were loaded and separated by 10% SDS-PAGE. The proteins were transferred onto 0.45-μm polyvinylidene fluoride membranes (#IPVH00010 from Merck). The membranes were blocked with 5% (w/v) skim milk and subsequently incubated with various primary antibodies overnight at 4 °C. After washing, the membranes were hybridized with a horseradish peroxidase-conjugated goat anti-rabbit IgG (#SA00001-2 from Proteintech, Wuhan, Hubei Province, China) for 1 h at room temperature, and the protein bands were visualized using Immobilon Western Chemiluminescent HRP Substrate (#WBKLS0500 from Merck). Digital images were taken with a chemiluminescence imaging system (Model 4800) from Tanon (Shanghai, China). Band intensities were quantified using ImageJ software from the National Institutes of Health (Bethesda, Maryland, United States), and the expression levels of target proteins were normalized to β-actin. The following primary antibodies were used: anti-STAT1 (#10144-2-AP) was from Proteintech; anti-JAK1 (#A11963) and anti-STAT6 (#A19120) were from ABclonal; and anti-β-actin (#ab8227) was from Abcam (Cambridge, United Kingdom).

### 2.5 Measurement of mRNA stability

THP-1 cells were seeded in 12-well plates (4 × 10^5^ cells per well in 1 mL culture media). Cells were pre-stimulated with 1 μg/mL of LPS for 12 h. Cells were then divided into two treatment groups: actinomycin D (10 μg/mL) plus DMSO (0.1% as vehicle control) and actinomycin D plus And (10 μM). Samples were harvested at 0, 2, 4, 6, 8, and 10 h after treatment. The mRNA levels of STAT1 were quantified using real-time PCR as described above.

### 2.6 Luciferase reporter assay

The luciferase reporter plasmid containing ISRE (ISRE-Luc) was purchased from Agilent Technologies (#219092, Santa Clara, California, United States). The reporter plasmid containing nuclear factor (NF)-κB binding motif (NF-κB-Luc) was obtained from Addgene (#185695, Watertown, Massachusetts, United States). HEK-293T cells were seeded in 12-well plates and cultured to be ∼70% confluent. Plasmid transfection was performed using Lipofectamine 3000 reagent (#L3000015 from Thermo Fisher) diluted in serum-free Dulbecco’s modified Eagle medium according to the manufacturer’s instructions. The final concentration of plasmid DNA in each well was 1 μg/mL, and the ratio of DNA to Lipofectamine was 2:3 (µg/µL). The cells were incubated with the DNA–Lipofectamine complex for 6 h, after which the medium was replaced with fresh medium, and the cells were further cultured overnight. The cells were then stimulated with 20 ng/mL IFN-β (for ISRE-Luc) or 10 ng/mL TNF-α (for NF-κB-Luc) for various durations, as indicated. The luciferase activities in cell lysates were measured using an assay kit (#E1910) from Promega (Madison, Wisconsin, United States) according to the manufacturer’s protocol. The chemiluminescent signal was detected using a microplate reader (Infinite E Plex from TECAN, Männedorf, Switzerland).

### 2.7 Measurement of nitric oxide release

Nitric oxide (NO) release was quantified by measuring the levels of nitrite in the cell culture medium using a colorimetric Nitric Oxide Assay Kit (#A012, from Jiancheng Bioengineering Institute, Nanjing, Jiangsu Province, China). Absorbance at 550 nm was measured using the Tecan plate reader.

### 2.8 Measurement of reactive oxygen species

Reactive oxygen species (ROS) production was measured using 2′,7′-dichlorodihydrofluorescein diacetate staining as described (Li et al., 2024). Cells were washed with PBS and incubated with 2′,7′-dichlorodihydrofluorescein diacetate (10 µM dissolved in phenol red-free culture medium) (#S0034S, from Beyotime, Shanghai, China) at 37 °C in the dark for 30 min. Fluorescent images were taken using a fluorescence microscope (model BX53F, Olympus, Tokyo, Japan). The mean fluorescence intensity was quantified using ImageJ software (NIH).

### 2.9 Statistical analysis

All data were expressed as the mean ± standard deviation (SD). Prism software (from GraphPad, San Diego, CA, United States) was used for statistical analyses. One-way analysis of variance (ANOVA), followed by *post hoc* Tukey’s test, was used for multiple comparisons. In some cases, data from particular two groups were also analyzed with an unpaired *t*-test, as explicitly reported in the figure legends. Two-tailed *p-*values of <0.05 were considered statistically significant. The dose-response curves were constructed using the nonlinear regression curve fitting function of the software. The analysis employed the “Dose-response–Inhibition” model with the “log (inhibitor) vs. normalized response–Variable slope” equation.

## 3 Results

### 3.1 Characterization of STAT1/2 mRNA expression in activated THP-1 cells

**FIGURE 1:**
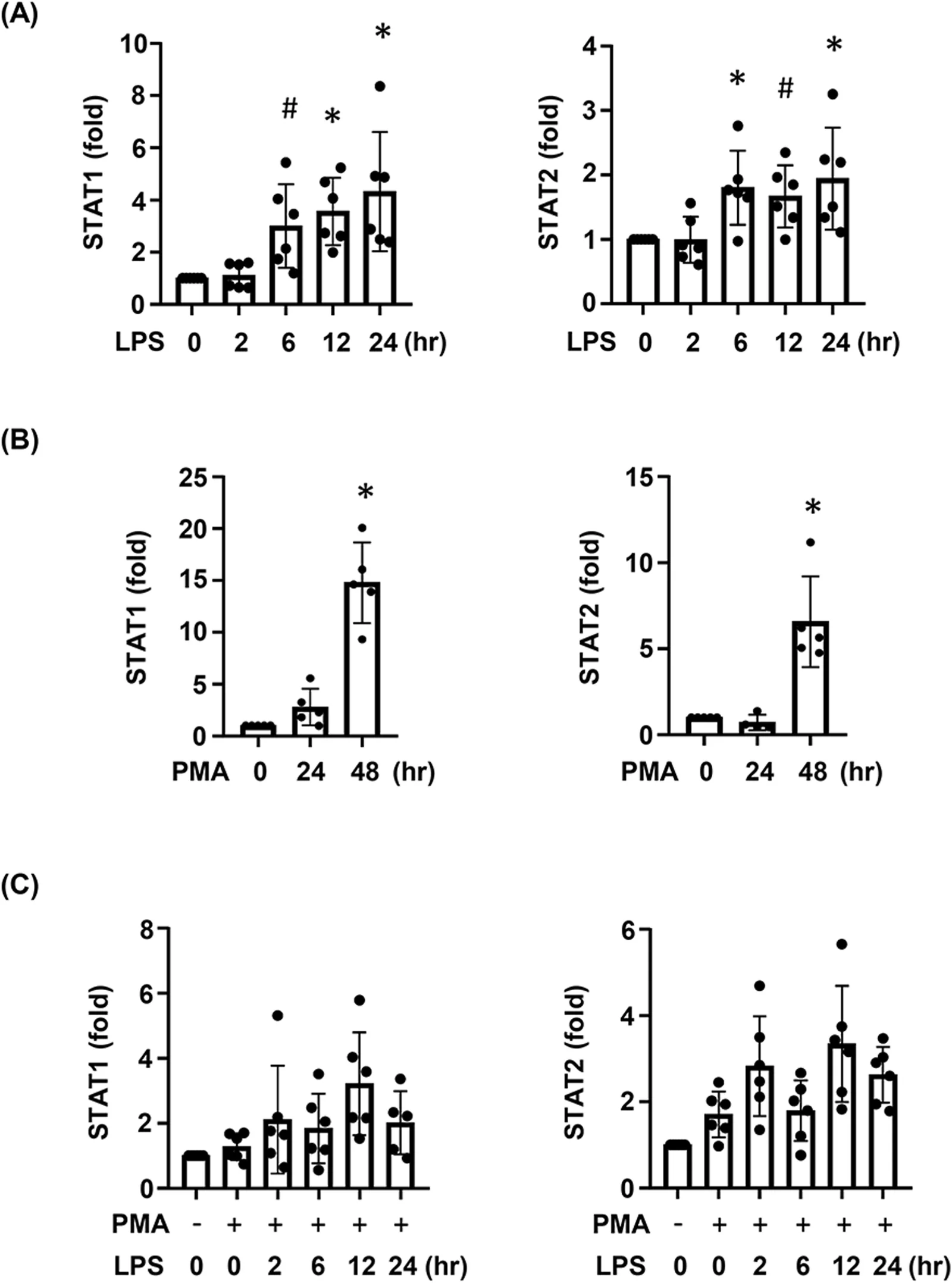
Real-time PCR results showing the time course of STAT1/2 expressions in activated THP-1 cells. Cells were stimulated with **(A)** LPS (1 μg/mL), **(B)** PMA (100 ng/mL), or **(C)** LPS plus PMA for various durations as indicated. Data were expressed as mean ± SD. ^*^ *P* < 0.05 versus control (0 hr) by one-way ANOVA; ^#^ *P* < 0.05 versus control by unpaired *t*-test. Each dot represented an independent experiment.

We first characterized the patterns of STAT1/2 expression in stimulated THP-1 cells. Treatment with LPS in undifferentiated THP-1 cells significantly increased the expression of both STAT1 and STAT2 in a time-dependent manner within 24 h (Figure 1A). Treatment of the cells with PMA, which is used to induce differentiation of THP-1, also upregulated STAT1 and STAT2 expression at 48 h (Figure 1B). However, no synergistic effects of PMA and LPS co-treatment on STAT1/2 expressions were observed (Figure 1C). Therefore, in the following experiments, we tested the effects of AP constituents in cells stimulated with LPS (24 h) or PMA (48 h) separately.

### 3.2 And, but not Neo or Deo, inhibited STAT1/2 expression

**FIGURE 2:**
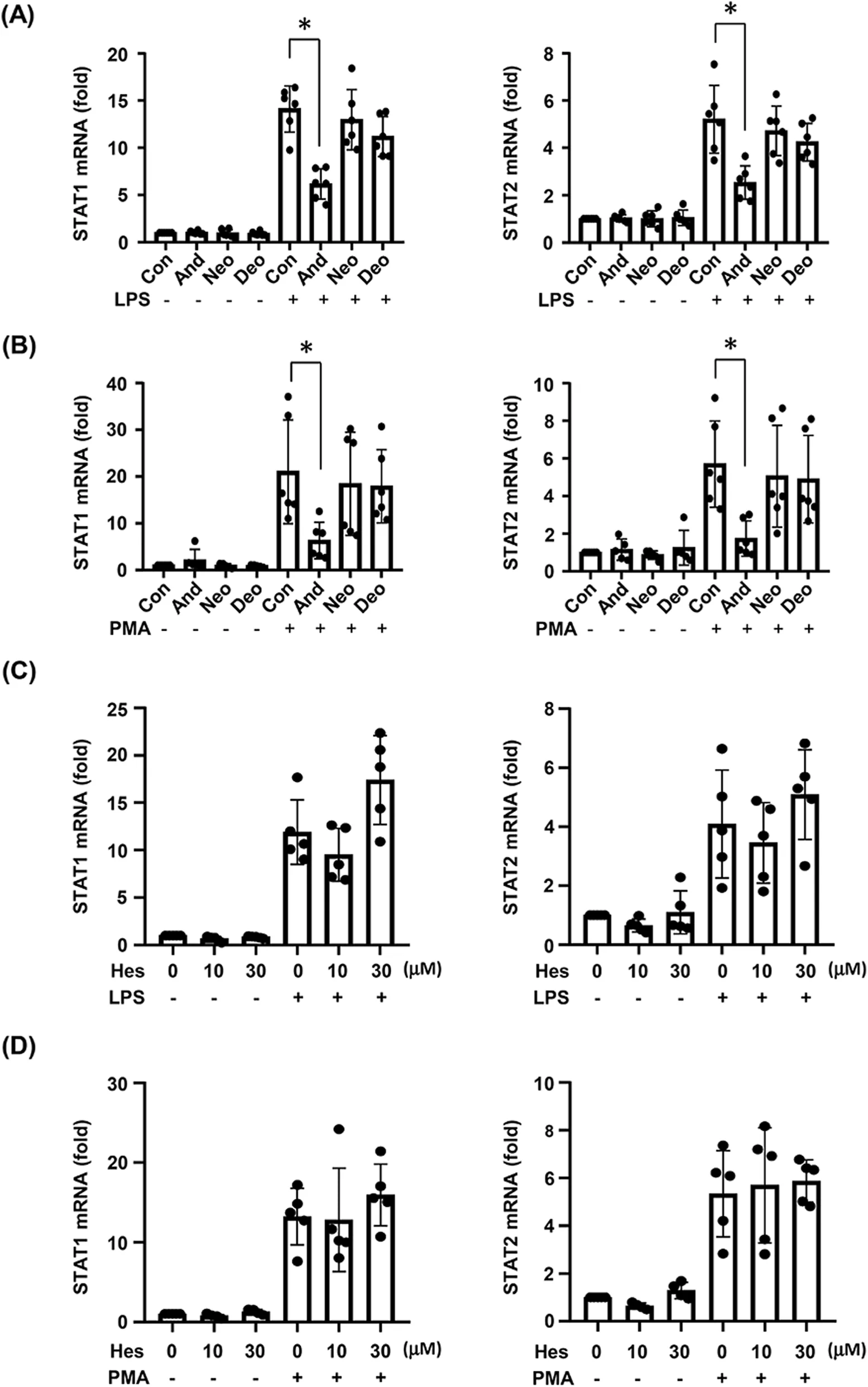
Real-time PCR results showing the effects of **(A,B)** And, Neo, Deo, and **(C,D)** hesperidin pre-treatments on STAT1/2 expressions in THP-1 cells, in the absence and presence of **(A,C)** LPS (1 μg/mL for 24 hr) or **(B,D)** PMA (100 ng/mL for 48 hr). And, Neo and Deo were all at 10 μM. Data were expressed as mean ± SD. ^*^ *P* < 0.05, one-way ANOVA. Each dot represented an independent experiment.

Cells were pre-treated with And, Neo, or Deo (all at 10 μM) for 2 h, then stimulated with LPS or PMA. We found that And significantly reduced both LPS- and PMA-induced STAT1/2 expressions (Figures 2A,B). In contrast, these effects were not observed with Neo or Deo (Figures 2A,B). To verify the specificity of our experimentation, we also tested the effects of hesperidin, an unrelated natural compound with anti-inflammatory and antioxidant activities (Tejada et al., 2018). We found that, as Neo and Deo, hesperidin (at 10 or 30 µM) did not exhibit significant effects on LPS- or PMA-stimulated STAT1/2 expressions (Figures 2C,D).

### 3.3 Pharmacodynamics of And-induced inhibition on STAT1/2 expression

**FIGURE 3:**
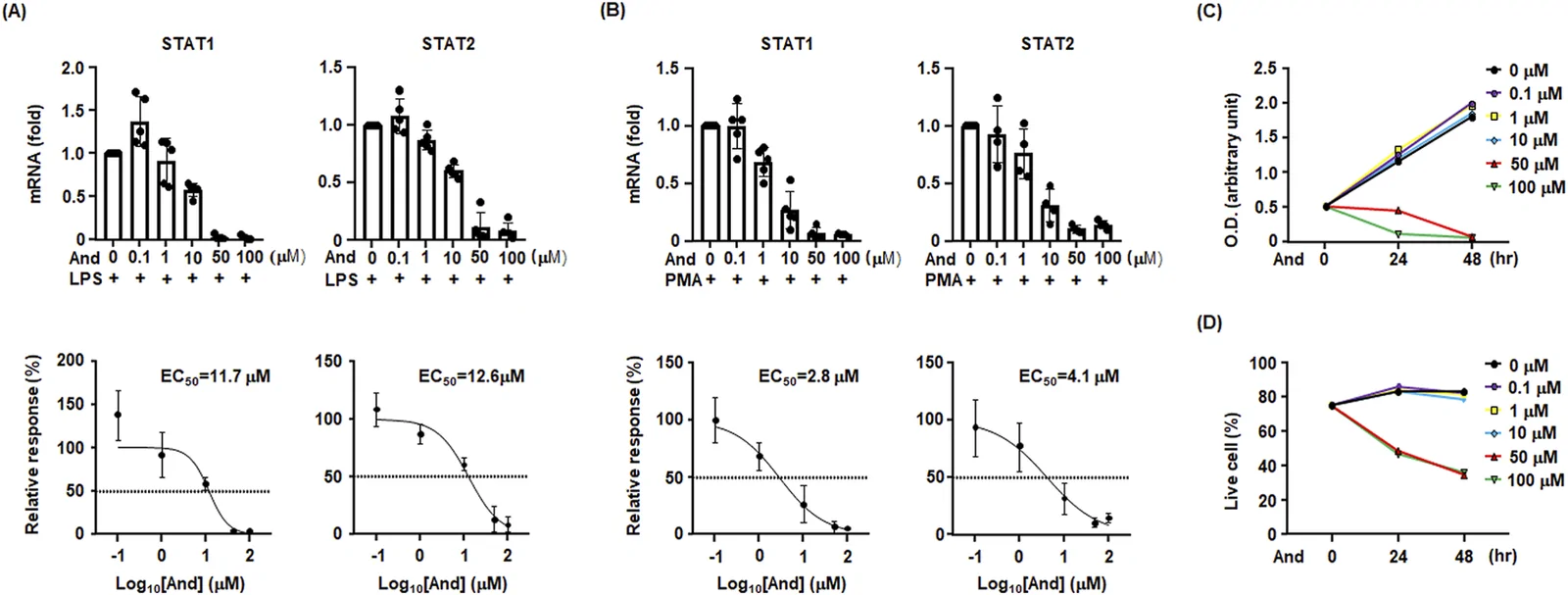
Characterization of the pharmacodynamics of And-induced inhibition on STAT1/2 expressions in activated THP-1 cells. **(A,B)** Real-time PCR results showing the concentration-dependent effects of And pre-treatment in cells stimulated with LPS (1 μg/mL for 24 hr) or PMA (100 ng/mL for 48 hr) respectively. Each dot represented an independent experiment. The corresponding dose-response curves and calculated EC_50_ values were shown below the bar graphs. **(C,D)** Cytotoxic effects of And in THP-1 cells determined with CCK-8 assay and Trypan Blue exclusion assay respectively (*n* = 5 independent experiments). Data were expressed as mean ± SD.

To further characterize the pharmacological effects of And on STAT1/2 expression, we constructed full concentration–response curves using And concentrations of 0.1–100 µM. In LPS-stimulated cells, we determined that the EC_50_ values of the inhibitory effects of And on STAT1 and STAT2 expression were 11.7 and 12.6 µM (Figure 3A). In PMA-stimulated cells, EC_50_ values were 2.8 and 4.1 µM, respectively (Figure 3A). To be of any value in therapeutic applications, it is essential to establish that the effective concentrations of the compound are devoid of cytotoxicity. For this purpose, we performed two different cytotoxicity assays. As shown in Figures 3C,D, both CCK-8 and Trypan Blue exclusion assays demonstrated that And of up to 10 µM had little effect on cell viability. Therefore, we used 10 µM of And for further experiments. Next, we examined whether post-treatment with And could reverse the upregulated STAT1/2 expression in pre-activated cells. THP-1 cells were stimulated with LPS for 12 or 24 h, followed by co-treatment with And for an additional 24 h. As shown in Supplementary Figure S1, we found that post-treatment with And did not reverse the upregulated STAT1/2 expressions in pre-activated macrophages.

### 3.4 And reduced the protein levels of STAT1

**FIGURE 4:**
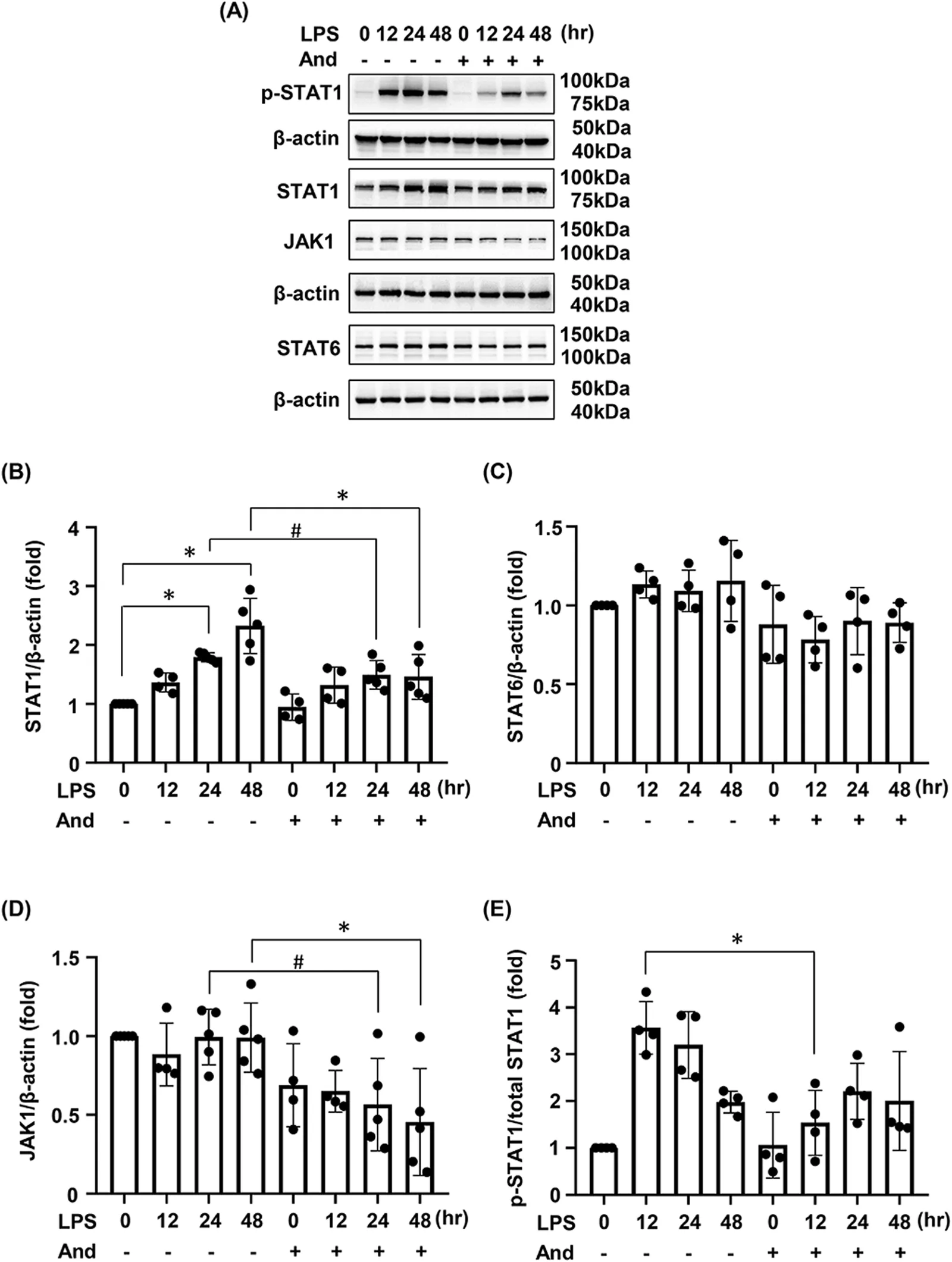
Western blotting analysis results showing the effects of And (10 μM) on the protein expression levels of phosphorylated STAT1 (p-STAT1), total STAT1, STAT6, and JAK1 in LPS (1 μg/mL)-stimulated THP-1 cells. **(A)** Representative original western blot images. **(B–E)** Quantitative relative densitometry data. Data were expressed as mean ± SD. ^*^ *P* < 0.05 by one-way ANOVA; ^#^ *P* < 0.05 by unpaired *t*-test. Each dot represented an independent experiment.

To confirm the inhibitory effect of And on STAT1 expression observed in the above PCR experiments, we performed Western blotting in order to clarify the effect of And on the protein level of STAT1. It was demonstrated that LPS stimulation time-dependently increased the level of STAT1 protein, while And pre-treatment significantly reduced the STAT1 expression (Figures 4A,B). In comparison, we showed that And had no effect on the expression of STAT6, which was not responsive to LPS stimulation either (Figure 4C). Moreover, we showed that the expression of JAK1 was not changed by LPS, while the effect of And on JAK1 was inconsistent and not statistically significant (Figure 4D). Finally, we measured the levels of phosphorylated STAT1 and showed that, in addition to the reduction in total STAT1, And treatment further decreased the level of phosphorylated STAT1 in LPS-challenged cells at 12 h (Figure 4E).

### 3.5 And inhibited the priming effects of IFN-γ

**FIGURE 5:**
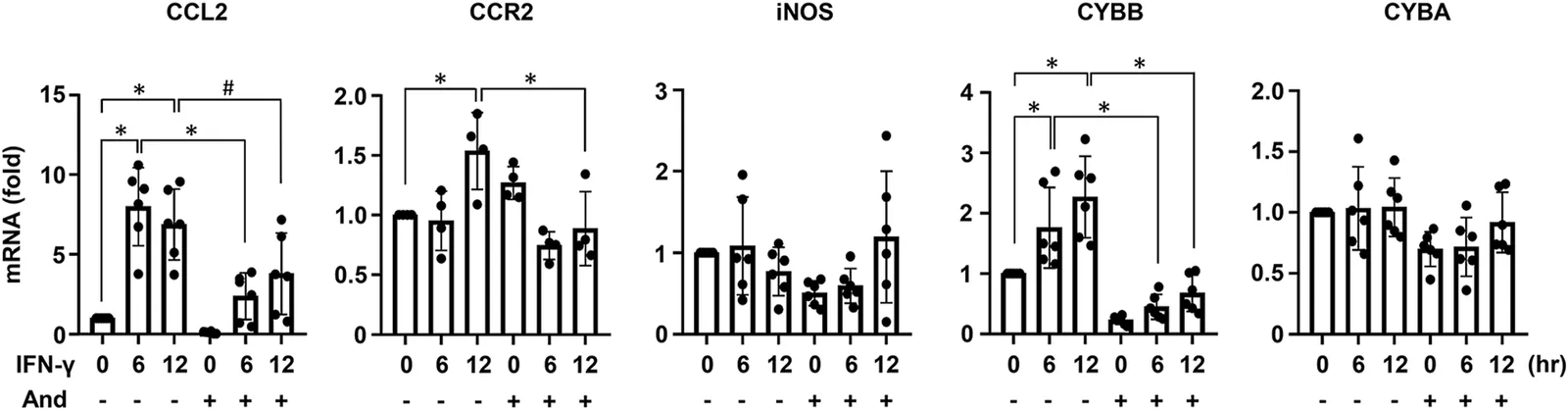
Real-time PCR results showing the effects of And pre-treatment on IFN-γ-induced expression of various target genes in THP-1 cells. Cells were pre-stimulated with PMA (100 ng/mL) alone or with And (10 μM) for 48 hr before addition of IFN-γ (20 ng/mL). Data were expressed as mean ± SD. ^*^ *P* < 0.05 by one-way ANOVA; ^#^ *P* < 0.05 by unpaired *t*-test. Each dot represented an independent experiment.

To test whether And-induced inhibition of STAT1 expression may repress the pro-inflammatory priming effects of IFN-γ in macrophages, we incubated THP-1 cells with PMA alone or PMA + And for 48 h, then stimulated the cells with IFN-γ for 6 or 12 h. We selected several macrophage-characteristic pro-inflammatory genes and performed PCR assays. As shown in Figure 5, IFN-γ upregulated the expression of CCL2 (C-C motif chemokine ligand 2, aka monocyte chemoattractant protein-1/MCP-1) and CCR2 (C-C motif chemokine receptor 2), while And pre-treatment significantly blunted the effects of IFN-γ. Unexpectedly, we did not observe any changes in the expression of iNOS in response to either IFN-γ or And. CYBB (cytochrome b-245 beta chain, aka gp91phox), a subunit of the phagocytic NADPH oxidase, is a well-documented IFN-γ target gene (Cassatella et al., 1990). We showed that IFN-γ upregulated the expression of CYBB, a response that was inhibited by And (Figure 5). In contrast, neither IFN-γ or And changed the expression of CYBA (cytochrome b-245 alpha chain, aka p22phox), another NADPH oxidase subunit that was not an IFN-γ target (Cassatella et al., 1990).

### 3.6 Mechanism of And-induced inhibition on STAT1 expression

**FIGURE 6:**
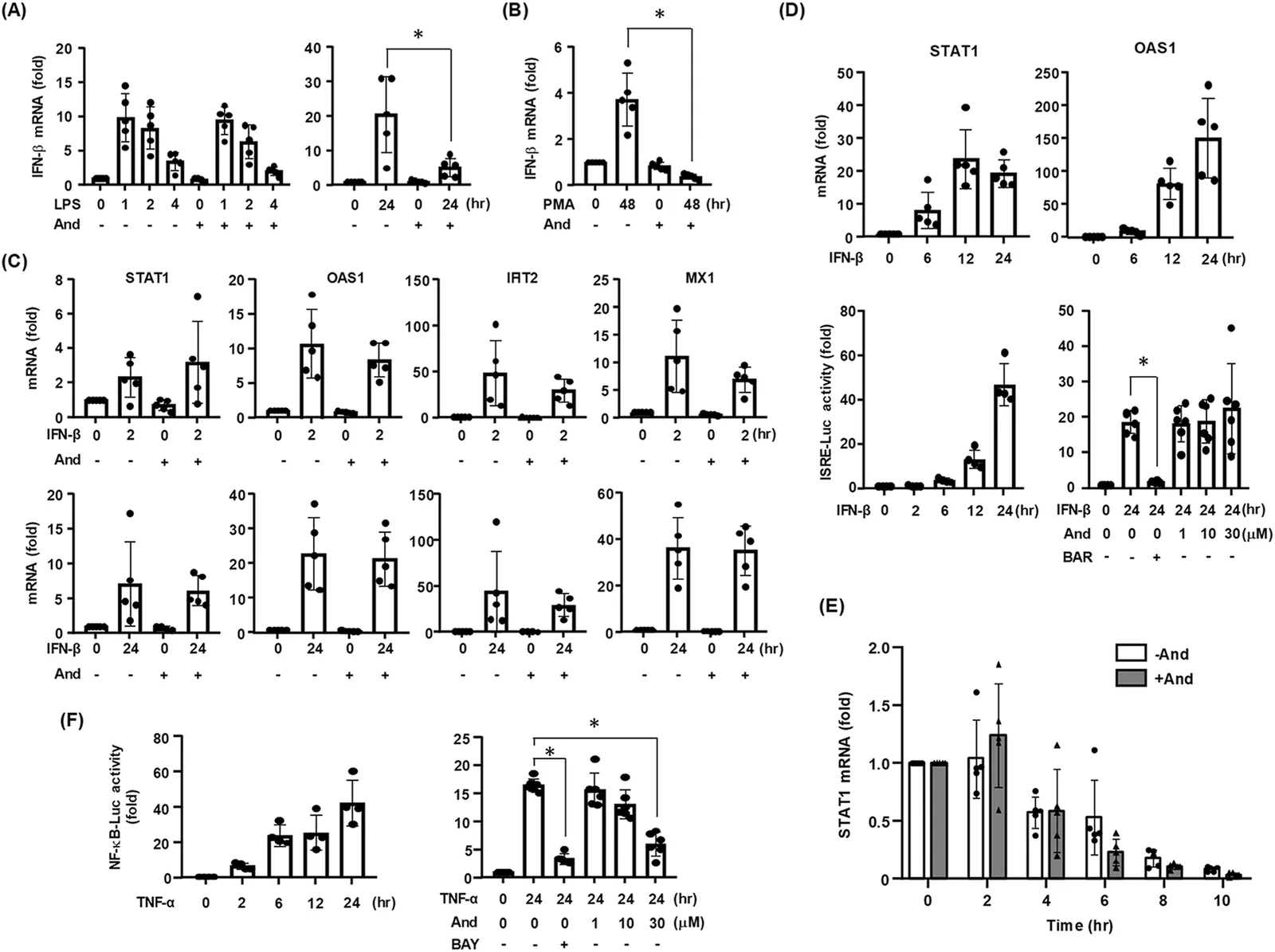
Mechanism of And-induced inhibition on STAT1 expression. **(A,B)** Real-time PCR results showing the effects of And (10 μM) on IFN-β expression in THP-1 cells stimulated with LPS (1 μg/mL) or PMA (100 ng/mL) respectively. **(C)** PCR results showing the effects of And on IFN-β (20 ng/mL)-induced expressions of ISGs in THP-1 cells at different time points. **(D)** The effect of And on IFN-β (20 ng/mL)-induced activation of ISGF3 tested in HEK-293T cells. The upper two panels were PCR results showing that IFN-b treatment robustly induced the expression of STAT1 and OAS1 in HEK-293T cells in a time-dependent manner. The lower two panels were luciferase reporter assay results showing that IFN-β time-dependently stimulated ISGF3 activation (left) while this response was not changed by And but was abolished by the JAK1/2 inhibitor baricitinib (BAR) (1 μM). **(E)** The effect of And on the rate of decay of STAT1 mRNA in LPS-activated THP-1 cells measured in the presence of actinomycin D (10 μg/mL). **(F)** Luciferase reporter assay results showing the effect of And on TNF-α (10 ng/mL)-induced activation of NF-κB tested in HEK-293T cells. The left panel showed that TNF-α stimulated NF-κB activation in a time dependent manner. The right panel showed the effects of And and the IκB kinase inhibitor BAY 11-7082 (10 μM) on TNF-α-induced NF-κB activation. Data were expressed as mean ± SD. ^*^ *P* < 0.05 by one-way ANOVA. Each dot represented an independent experiment.

In macrophages, the expression of STAT1 is primarily driven by type I IFN signaling (Amalraj et al., 2013; Yuasa and Hijikata, 2016). Based on this notion, we first examined whether And regulates IFN-β expression in stimulated THP-1 cells. As shown in Figure 6A, LPS robustly induced IFN-β expression within 2 h, while the expression level declined nearly to the baseline at 4 h. This finding is consistent with previous reports showing that the response of LPS-induced macrophage IFN-β expression is transient (Jacobs and Ignarro, 2001; Thomas et al., 2006). We showed that And had no effect on this initial IFN-β expression following LPS stimulation (Figure 6A). However, in contrast to the declined IFN–β level at 4 h, we found that the level of IFN-β was significantly elevated at 24 h, which might represent a second (sustained) wave (Mulvey et al., 2021) of type I IFN expression (Figure 6B). Notably, And pre-treatment reduced the IFN-β expression at 24 h (Figure 6B). The reduced production of IFN-β in And-treated cells was consistent with the observed decrease in STAT1 phosphorylation (see Figure 4E). Moreover, we observed And also exhibited a similar inhibitory effect on IFN–β expression in PMA (48 h)-treated cells (Figure 6B).

The above data indicated that the inhibitory effects of And on STAT1 expression could be explained, at least partly, by reduced IFN-β production. To elucidate whether And had any direct effects on type I IFN-driven STAT1 transcription, we treated the cells with recombinant IFN-β for 2 or 24 h. At both time points, IFN-β significantly increased the mRNA levels of STAT1; however, And exhibited no effects on IFN-β-stimulated STAT1 expression (Figure 6C). To confirm the ineffectiveness of And on IFN-β-induced transcription, we also measured the mRNA levels of several typical ISGs, including OAS1 (2′-5′-oligoadenylate synthetase 1), IFIT2 (interferon-induced protein with tetratricopeptide repeats 2), and MX1 (MX dynamin-like GTPase 1). We showed that And did not change the mRNA expression of these genes either (Figure 6C). Furthermore, we examined whether And had any effect on the function of transcription factor ISGF3. For this purpose, we used the HEK-293T cell line since plasmid transfection was technically difficult in THP-1 cells. We demonstrated that IFN-β treatment of HEK-293T cells significantly upregulated the expression of STAT1 and OAS1 (Figure 6D), confirming that this cell line contains all functional elements of the canonical type I IFN signaling (Kim et al., 2023). Then, we transfected HEK-293T cells with ISRE-Luc reporter plasmids and performed luciferase assays. We showed that IFN-β treatment for 2–24 h increased the ISGF3 activity in a time-dependent manner (Figure 6D). Nevertheless, And had no effect on IFN-β-induced ISGF3 activation, which was abolished by the selective JAK1/2 inhibitor baricitinib (Figure 6D). To determine whether And modulated the stability of STAT1 mRNA, we treated the cells with actinomycin D to block *de novo* mRNA synthesis and measured the rate of STAT1 mRNA decay. As shown in Figure 6E, And treatment did not change the stability of STAT1 mRNA. It is not clear how And inhibits the expression of IFN-β. There is evidence suggesting that NF-κB is involved in facilitating IFN-β transcription in addition to IRF3/7 (Honda et al., 2006), and And has an inhibitory effect on NF-κB (Xia et al., 2004). Hence, we examined whether And modified the activity of NF-κB in HEK-293T cells transfected with NF-κB-Luc plasmids. Treatment with TNF-α time-dependently increased the NF-κB activity (Figure 6F). This response was abolished by the selective IκB kinase inhibitor BAY 11-7082. And significantly inhibited TNF-α-induced NF-κB activation only at 30 µM (Figure 6F).

### 3.7 Effects of And in primary macrophage cells

**FIGURE 7:**
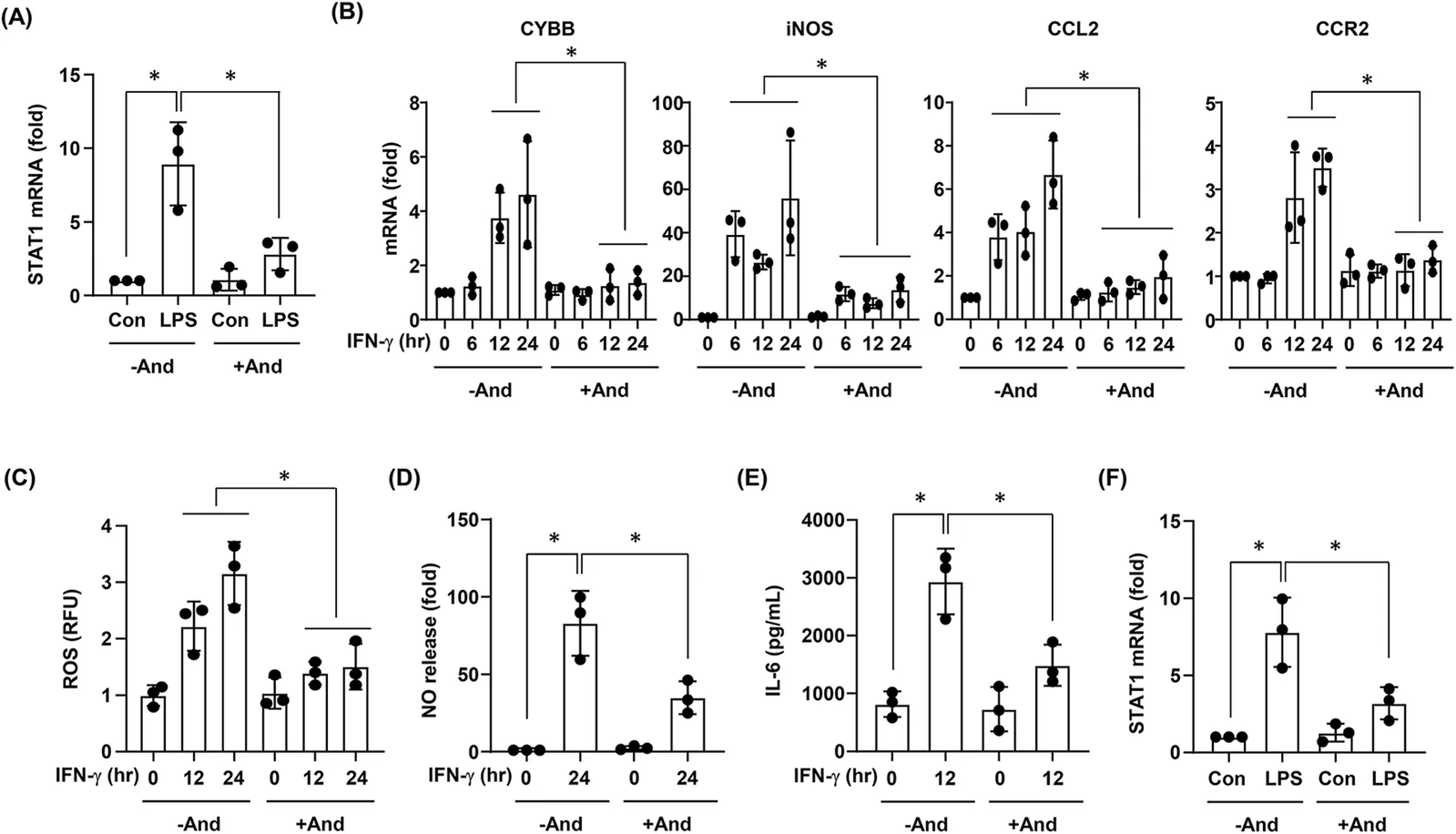
Effects of And in primary murine bone marrow cell-derived (panels A to E) and human peripheral blood mononuclear cell-derived (panel F) macrophages. **(A)** PCR results showing the effect of And (10 μM) co-treatment on the expression of STAT1 in LPS (100 ng/mL for 24 hr)-stimulated cells. **(B)** PCR results showing the effects of And pre-treatment on IFN-γ (20 ng/mL)-induced priming in LPS-preactivated cells (And added and washed off together with LPS). **(C,D)** Effects of And pre-treatment on ROS production and NO release in response to IFN-γ (20 ng/mL) in LPS-preactivated cells (And added and washed off together with LPS). **(E)** ELISA results showing the effect of And pre-treatment on LPS-driven IL-6 secretion. Cells were preactivated with LPS with and without And for 12 hr. Cells were washed and primed with IFN-γ with and without And for 12 hr. Cells were washed again and re-challenged with LPS alone for 8 hr. **(F)** Real-time PCR results showing the effect of And (10 μM) co-treatment on STAT1 expression in human primary macrophages activated by LPS (100 ng/mL for 24 hr). Data were expressed as mean ± SD. ^*^ *P* < 0.05, one-way ANOVA. Each dot represented an independent experiment.

In order to further establish the inhibitory effects of And on STAT1 expression and function, we repeated several key experiments in murine and human primary macrophages. First, we demonstrated that in LPS-stimulated murine bone marrow cell-derived macrophages, the expression of STAT1 was significantly reduced by And (Figure 7A); this effect was consistent with that observed in THP-1 cells. Next, we confirmed the inhibitory effects of And on IFN-γ-induced priming in murine primary macrophages. Specifically, we showed that in cells pre-activated by LPS, co-treatment with And significantly attenuated IFN-γ-induced expression of CYBB, CCL2, and CCR2 (Figure 7B). Unlike the response in THP-1 cells, we found that IFN-γ also induced iNOS expression. Similarly, this IFN-γ response was repressed by And (Figure 7B). To clarify whether the inhibitory effects of And on mRNA expression reflected functional changes in the macrophage cells, we measured the production of reactive oxygen species (ROS) (attributable to CYBB induction) and NO (attributable to iNOS induction) in murine macrophages. As shown in Figures 7C,D, And treatment significantly reduced the production of ROS and NO in response to IFN-γ. To further demonstrate the functional importance of the anti-priming effects of And, we measured the secretion of IL-6. In LPS-preactivated murine macrophages, priming by IFN-γ enhanced the secretion of IL-6 driven by a second round of LPS stimulation (Figure 7E). Co-treatment with And significantly reduced the production of IL-6 (Figure 7E). However, we found that And had little effect in cells without IFN-γ priming. We reasoned that this phenomenon was due to the fact that LPS-induced IL-6 expression largely relied on NF-κB (Baccala et al., 2009), while our data indicated that And only had a relatively weak effect on NF-κB function (see Figure 6F). To corroborate this argument, we performed additional PCR assays and confirmed that in cells stimulated with LPS alone, the upregulation of IL-6 expression was not changed by And (Supplementary Figure S2). Nonetheless, these results further supported the notion that the anti-inflammatory effects of And observed under the present experimental settings were a consequence of the inhibition of STAT1-mediated signaling. For this reason, we finally tested whether And was able to inhibit STAT1 expression in human primary macrophages. As shown in Figure 7F, And co-treatment significantly decreased STAT1 expression in LPS-stimulated human peripheral blood mononuclear cell-derived macrophages. To take part in the Resource Identification Initiative, please use the corresponding catalog number and RRID in your current manuscript. For more information about the project and steps on how to search for an RRID, please click here.

## 4 Discussion

In the present study, we identified that the major bioactive compound And from *A. paniculata* inhibited the expression of STAT1 in both LPS- and PMA-stimulated macrophage cells. The effective concentration for this action was approximately 10 μM, which exhibited no cytotoxicity. No such effect was observed with two related diterpenoid derivatives, Neo or Deo, or an unrelated natural compound, hesperidin. It is believed that the structural differences between And and Neo/Deo determine their unequal efficacies, although the precise mechanisms remain to be elucidated. Nevertheless, the ineffectiveness of Neo and Deo compared to And was also observed in other biological functions (Theerawatanasirikul et al., 2022; Lu et al., 2025). STAT1 is a pivotal transducer in type I IFN signaling; concomitantly, STAT1 is also an effector (target gene) of type I IFN signaling, forming a feedforward loop (Ivashkiv and Donlin, 2014; McNab et al., 2015). The biological effects of type I IFNs in macrophages are implicated in LPS-induced systemic inflammation and lethality (Karaghiosoff et al., 2003; Thomas et al., 2006; Kelly-Scumpia et al., 2010). Overactivation of the type I IFN signaling following viral infections may cause immunopathology by stimulating inflammatory reactions in macrophages (McNab et al., 2015; Channappanavar et al., 2016; Domizio et al., 2022). On the other hand, STAT1 is also essential in mediating the pro-inflammatory (M1) macrophage-priming effects of INF-γ (Hu et al., 2002; Gough et al., 2010; Ivashkiv, 2018). Aberrant activation of the IFN-γ pathway underlies several, potentially fatal, hyperinflammatory or immune-mediated diseases (De Benedetti et al., 2021). Therefore, our results suggest that, under some pathological conditions, repressing STAT1 expression by And may produce beneficial anti-inflammatory effects, preventing the development of systemic inflammatory response syndrome (Castellheim et al., 2009). We argue that this effect of And may, at least partly, explain the multiple anti-inflammatory effects of the compound observed in various preclinical models (Shan et al., 2025). Particularly, our data are partly supported by those from another group (Ding et al., 2017), showing that And markedly inhibited the expression of STAT1 in influenza A virus-infected macrophage cells.

PRR stimulation in monocytes/macrophages elicits the production of type I IFNs (López De Padilla and Niewold, 2016), which may, in turn, induce the expression of ISGs (including STAT1) in an autocrine manner (Sheikh et al., 2014). Among the type I IFN family members, IFN-β appears to have a predominant role in mediating the IFN response in LPS-challenged macrophages (Thomas et al., 2006; Sheikh et al., 2014). Because And did not change the responses induced by IFN-β, including target gene expressions and the activation of ISGF3 (as shown by the ISRE-Luc reporter assay), our data indicated that And had no effects on the signaling events downstream of IFN-β/IFNAR. Instead, our results suggested that And inhibited STAT1 expression by reducing IFN-β production. Notably, this indirect effect of And might explain the observation that post-treatment with And in pre-activated macrophages did not reverse the upregulated expression of STAT1, since once expressed, STAT1 (in an unphosphorylated status) could sustain the IFN-β-triggered transcriptional response even in the absence of IFN-β (Cheon et al., 2013). On the other hand, there is evidence suggesting that STAT2 expression is under the transcriptional control of STAT1 (Yuasa and Hijikata, 2016). Consistent with this notion, the inhibitory effect of And on STAT2 expression is likely to be secondary to its effect on STAT1 expression.

At present, it is not clearly understood how And regulates the expression of INF-β. We found that And had similar inhibitory effects on IFN-β expression in both LPS- and PMA-stimulated cells, which rely on distinct signal transduction mechanisms (TLR4–TRAF3–TBK1-dependent mechanisms and PKC-dependent mechanisms, respectively) (Johnson et al., 2007; Baccala et al., 2009). Hence, it is suggested that the And effect is unlikely to be due to specific interference with these proximal signaling modules. Upon TLR4 activation, INF-β expression is primarily driven by IRF3/7, with NF-κB acting as a co-activator (Honda et al., 2006). Interestingly, it was found that And did not change the initial INF-β expression following TLR4 activation but only significantly inhibited the delayed (sustained) phase of INF-β expression. First, these data excluded the possibility that And alters the functioning of the TLR4-IRF3/7 axis (Lim et al., 2015). Although we confirmed that And reduced the activity of NF-κB (Xia et al., 2004), this effect was relatively weak (occurring above 30 µM) under the present experimental settings, suggesting that inhibition of NF-κB was unlikely to have a major role in mediating the effects of And. We reasoned that the delayed phase of IFN-β may involve *de novo* protein expression of additional transcription co-activators. PMA may increase the expression of c-Jun, a prototype component of the transcription factor complex activating protein-1 (AP-1) (Lee et al., 2001), while c-Jun-containing AP-1 acts as a transcription co-activator in mediating IFN-β expression (Doly et al., 1998; Honda et al., 2006). Based on the above information, we propose that the mechanism(s) underlying And-induced inhibition of IFN-β expression may involve interactions with transcriptional regulators other than IRF3/7 and NF-κB (see Doly et al. (1998); Fan et al. (2023)). This outstanding question warrants further investigation.

In summary, we have identified that And inhibits STAT1 expression in activated macrophage cells. This effect desensitizes macrophages to IFN-γ-induced priming and may partly explain the multiple anti-inflammatory actions of this compound.

## Data Availability

The original contributions presented in the study are included in the article/Supplementary Material; further inquiries can be directed to the corresponding author.
