# Supplementary material for: Andrographolide inhibits STAT1 expression and desensitizes interferon γ-induced priming in macrophage cells
Source: Front Pharmacol. 2026 Sep 3;17:1855424. doi: 10.3389/fphar.2026.1855424 (PMC13581795; doi:10.3389/fphar.2026.1855424)
Supplement: Supplementary file 1 [file Image1.pdf]

Supplementary Figure S1

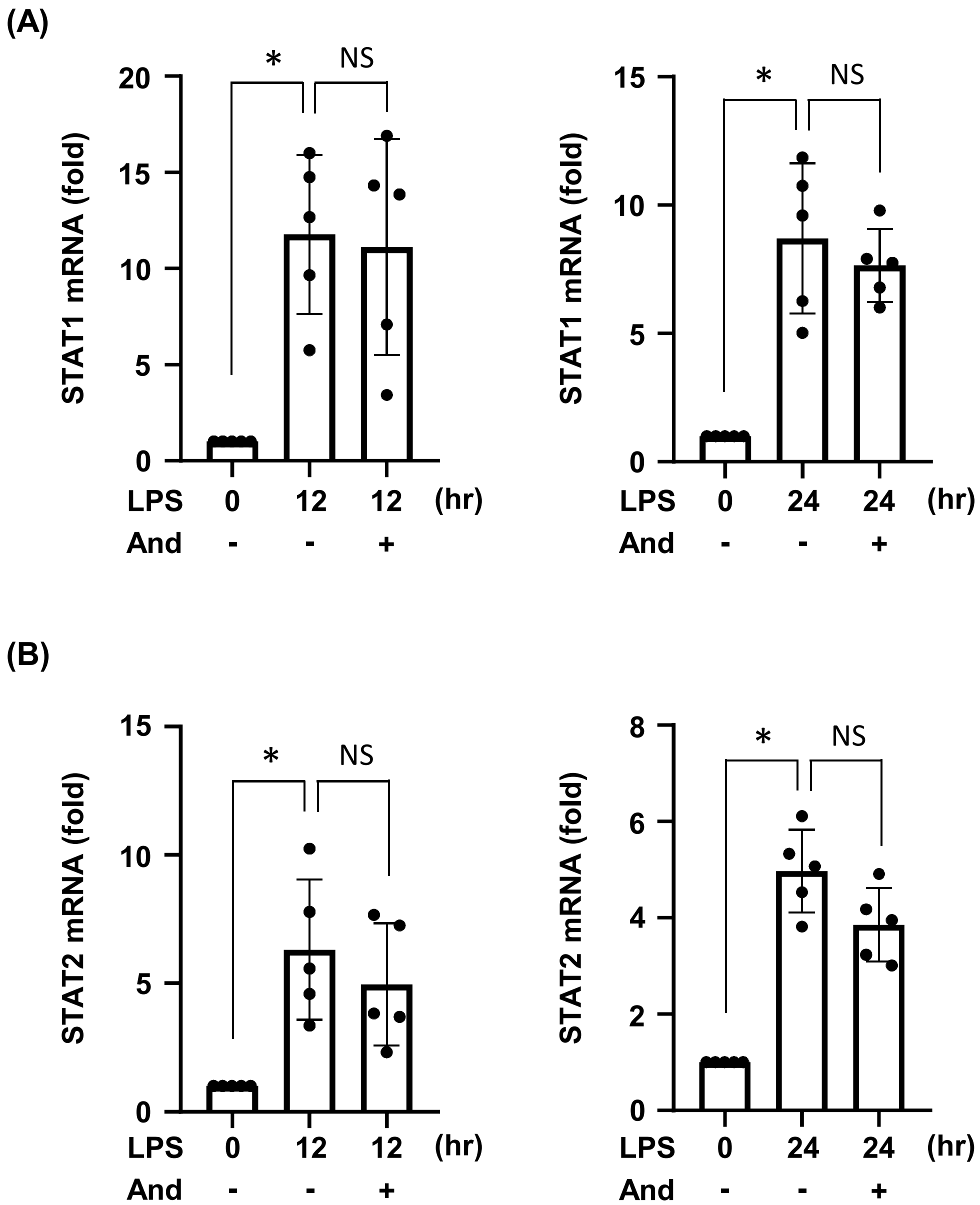

Figure S1. PCR results showing that post-treatment with And (10 μM) could not reverse the upregulated STAT1/2 expressions in pre-activated THP-1 cells. Cells were pre-stimulated with LPS for 12 or 24 hr, followed by co-treatment with And for additional 24 hr. Data were expressed as mean ±SD. * $P < 0.05$, one-way ANOVA; NS, no significance. Each dot represented an independent experiment.
