# Supplementary material for: Andrographolide inhibits STAT1 expression and desensitizes interferon γ-induced priming in macrophage cells
Source: Front Pharmacol. 2026 Sep 3;17:1855424. doi: 10.3389/fphar.2026.1855424 (PMC13581795; doi:10.3389/fphar.2026.1855424)
Supplement: Supplementary file 2 [file Image2.pdf]

Supplementary Figure S2

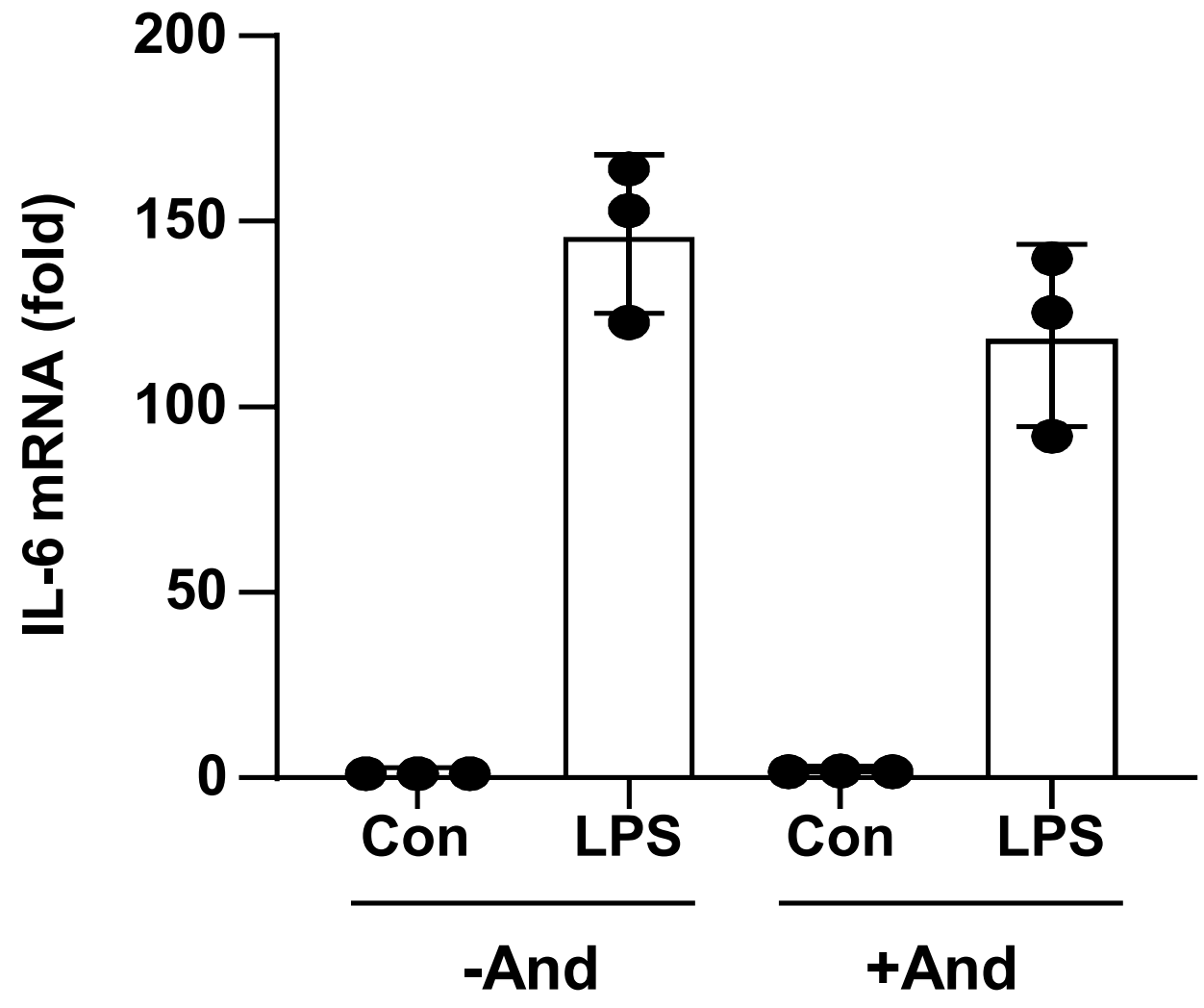

Figure S2. Real-time PCR results obtained in murine bone marrow cell-derived macrophages showing that And (10 μM) co-treatment had no significant effect on IL-6 expression stimulated by LPS (100 ng/mL for 4 hr) alone. Data were expressed as mean ± SD. Each dot represented an independent experiment.
