# Supplementary material for: Andrographolide inhibits STAT1 expression and desensitizes interferon γ-induced priming in macrophage cells
Source: Front Pharmacol. 2026 Sep 3;17:1855424. doi: 10.3389/fphar.2026.1855424 (PMC13581795; doi:10.3389/fphar.2026.1855424)
Supplement: Supplementary file 3 [file Image3.pdf]

Figure 4

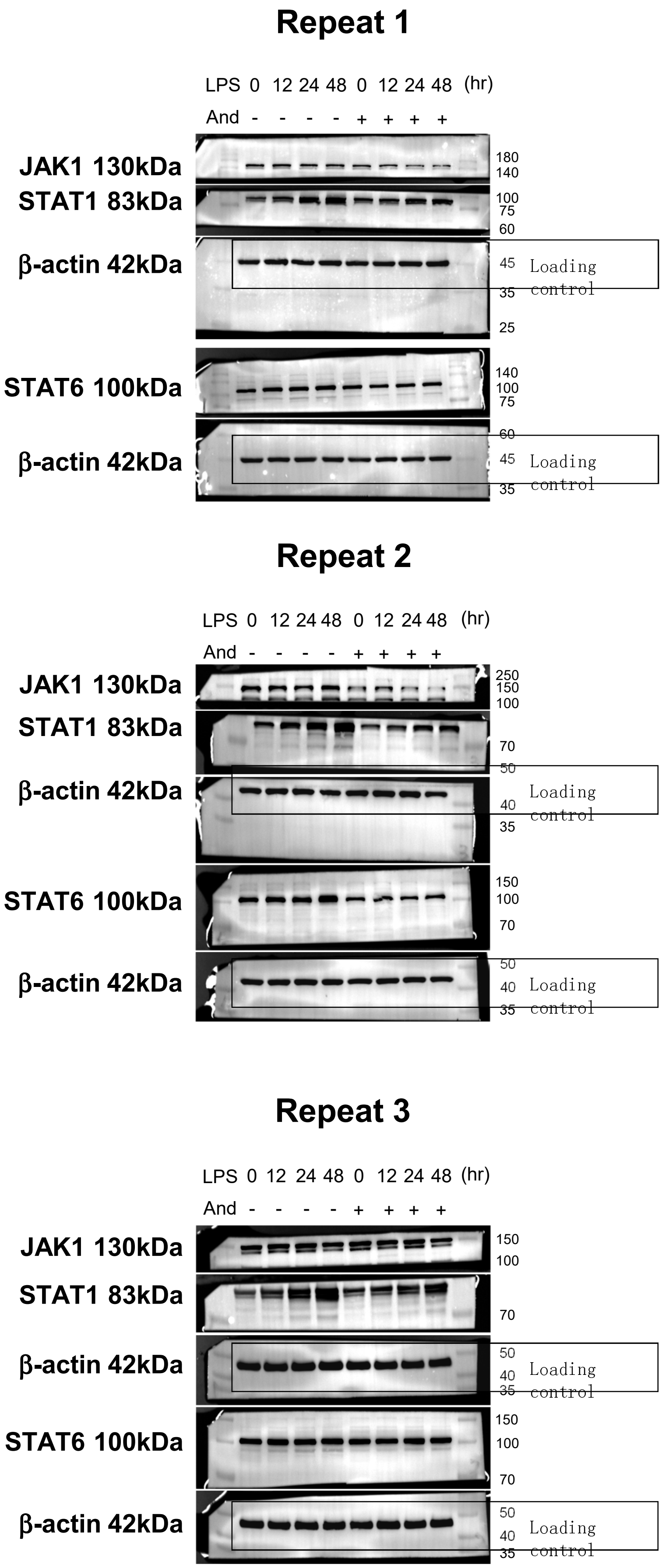
Repeat 1
LPS 0 12 24 48 0 12 24 48 (hr)
And - - - - + + + +
JAK1 130kDa
STAT1 83kDa
β-actin 42kDa
STAT6 100kDa
β-actin 42kDa
180
140
100
75
60
45
35
25
140
100
75
60
45
35
Loading control
Loading control
Repeat 2
LPS 0 12 24 48 0 12 24 48 (hr)
And - - - - + + + +
JAK1 130kDa
STAT1 83kDa
β-actin 42kDa
STAT6 100kDa
β-actin 42kDa
250
150
100
70
50
40
35
150
100
70
50
40
35
Loading control
Loading control
Repeat 3
LPS 0 12 24 48 0 12 24 48 (hr)
And - - - - + + + +
JAK1 130kDa
STAT1 83kDa
β-actin 42kDa
STAT6 100kDa
β-actin 42kDa
150
100
70
50
40
35
150
100
70
50
40
35
Loading control
Loading control
