# Supplementary material for: Andrographolide inhibits STAT1 expression and desensitizes interferon γ-induced priming in macrophage cells
Source: Front Pharmacol. 2026 Sep 3;17:1855424. doi: 10.3389/fphar.2026.1855424 (PMC13581795; doi:10.3389/fphar.2026.1855424)
Supplement: Supplementary file 4 [file Table1.pdf]

Supplementary Table S1. Primer sequences used for real-time PCR assays

Gene	Forward Primer (5'→3')	Reverse Primer (5'→3')
GAPDH	GCACCGTCAAGGCTGAGAAC	TGGTGAAGACGCCAGTGGA
STAT1	TGGGCCTCCATCCTTTGGTA	ACTGCCAACTCAGCACTTCT
STAT2	CGACCCCTCATCCTCAAGAC	CGTTGCTCCACCAGAGTCAG
CCL2	CTCAGCCAGATGCAATCAATG	CTTCTTTGGGACACTTGCTGC
CCR2	CCAACGAGAGCGGTGAAGAA	TGAACACCAGCGAGTAGAGC
NOS2	GCTTTGTGCGGAATGCCAG	CAAACACCAAGGTCATGCGG
CYBA	ACAGAAGTACATGACCGCCG	CAGCCGCCAGTAGGTAGATG
CYBB	GGGAACTGGGCTGTGAATGA	CCAGTGCTGACCCAAGAAGT
IFN-β	ACGCCGCATTGACCATCTAT	GTCTCATTCCAGCCAGTGCTA
OAS1	GGTCTATGCTTGGGAGCGAG	CAAGACCGTCCGAAATCCCT
IFIT2	TCGGCTGAATCCTGACAACC	TCGCTTTGTCTGGCTCATCT
MX1	GGATCGTGACCAGATGCCC	CTGATTCCCATTCCTTCCCCG
